# Automatically measuring brain ventricular volume within PACS using artificial intelligence

**DOI:** 10.1371/journal.pone.0193152

**Published:** 2018-03-15

**Authors:** Fernando Yepes-Calderon, Marvin D. Nelson, J. Gordon McComb

**Affiliations:** 1 Children’s Hospital Los Angeles, Division of Neurosurgery, Los Angeles, CA, United States of America; 2 Children’s Hospital Los Angeles, Department of Radiology, Los Angeles, CA, United States of America; 3 University of Southern California, Keck School of Medicine, Los Angeles, CA, United States of America; University of Pennsylvania, UNITED STATES

## Abstract

The picture archiving and communications system (PACS) is currently the standard platform to manage medical images but lacks analytical capabilities. Staying within PACS, the authors have developed an automatic method to retrieve the medical data and access it at a voxel level, decrypted and uncompressed that allows analytical capabilities while not perturbing the system’s daily operation. Additionally, the strategy is secure and vendor independent. Cerebral ventricular volume is important for the diagnosis and treatment of many neurological disorders. A significant change in ventricular volume is readily recognized, but subtle changes, especially over longer periods of time, may be difficult to discern. Clinical imaging protocols and parameters are often varied making it difficult to use a general solution with standard segmentation techniques. Presented is a segmentation strategy based on an algorithm that uses four features extracted from the medical images to create a statistical estimator capable of determining ventricular volume. When compared with manual segmentations, the correlation was 94% and holds promise for even better accuracy by incorporating the unlimited data available. The volume of any segmentable structure can be accurately determined utilizing the machine learning strategy presented and runs fully automatically within the PACS.

## 1 Introduction

The Picture Archiving and Communications System (PACS) is currently the standard platform to manage medical images [1] but lacks analytical and quantification capabilities [2, 3]. Staying within the PACS, the authors have developed an automatic method to retrieve the medical data and access it at a voxel level, decrypted and uncompressed that enables analytical procedures to be applied to the data while not perturbing the system’s daily operation. Additionally, the strategy is secure and vendor independent [4].

Being able to segment the cerebral ventricles to determine the quantity of cerebrospinal fluid (CSF) within the ventricles has widespread applicability in many neurological conditions. Although this segmentation would seem to be a relatively straightforward task, such is not the case requiring manual tracing of the ventricular outline on multiple image slices. This process is tedious, time-consuming, operator dependent and rarely done except for research purposes. It also requires IRB permission to move the images out of the PACS, anonymization, and format changing so the images are loadable by external software where the segmentations can be accomplished. Using the PACS vehicle presented [4], segmentations and other analytical tasks can be performed in the image storing system, provided that the procedures are fully automatic.

Brain ventricular segmentation has been approached with several methods that include registration-based techniques [5–7], fuzzy thresholding [8], seed-based for constant neighboring and boundary tracking [9, 10] and more recently artificial intelligence (AI) approaches that use voxel-based feature extraction in their core. Some of them require multi-modality images to refine results as in [11, 12], others use registration to atlases as in [13–16] to label regions prior to a classification stage. PACS related restrictions hinder the possibility of implementing the methods mentioned above due either to the need of human interaction and external image models as required by the template-based strategies or availability of multi-modality images. Additionally, some of the listed methods, despite being highly accurate with recruited data, have not been reported to work in randomly selected clinical data and may fail when dealing with clinical images that have restricted fields of view (FOV) or abnormalities.

Also, clinical imaging protocols and parameters are often varied to suit medical necessities and patient comfort, making it difficult to use a general solution with the standard segmentation techniques. As the contrast is variable, structures are harder to segment when small as the boundaries become less distinct. The segmentation solutions must also incorporate features other than those based solely on intensities. The presence of restricted FOV makes it necessary to work with a voxel-based technique. The method presented solves the segmentation issue by considering it as a classification problem. It utilizes a machine learning strategy whose features have been carefully chosen and optimized to reach a high level of accuracy without requiring external resources and running fully automatic within the PACS.

## 2 Materials and methods

### 2.1 Training and testing data in the creation of the support vector machine (SVM) estimator

With the aim of creating a robust estimator, 44 T1-contrast images –acquired at 1.5T– of patients treated at the hosting institution underwent the feature extraction procedure explained below in section 2.3. The process yielded a matrix with dimensions 53′065.316*x*6. The number of rows corresponds to the total number of voxels visited in the 44 studies after re-sampling each volume to 1*x*1*x*1 mm. Four of the columns hold the four features selected as separation magnifiers for this machine learning implementation, see section 2.3. One more column holds the supervising factor that defines whether a voxel belongs to the segmenting structure. This value is provided by manually segmented masks of the lateral ventricles. Experts perform the segmentation in the native spatial resolution profiting from the higher in-plane resolution of the original data respect to the 1*X*1 mm used in the presented ML exercise. The in-plane resolution of our clinical data is in the range of micrometers. After manual segmentation, all mask are re-sampled to 1*x*1*x*1 mm. Finally, the last column of the working matrix is used to keep a reference to the subject to which the voxel belongs.

In the manual segmentation activities an in-house developed software where collateral tasks such as DICOM image concatenation, image loading, sub-stacking, advancing along slices and mask saving, are performed automatically. The in-house developed software also accounts for human fatigue by turning off automatically after 30 minutes of working and disabling its use for 15 minutes. The operator, once authorized to return, is presented with the task precisely in the slice where the segmentation work was interrupted.

### 2.2 Clinical data in the use of the SVM estimator

The clinical data where segmentations are performed using the SVM estimator consisted of 10 magnetic resonance imaging (MRI) studies acquired with T1 contrast using a 1.5T Phillips Scanner. Five of those subjects were randomly collected. Three more subjects correspond to selected cases for mild, moderate and severe hydrocephalus (HC). The other two images correspond to a single subject with two imaging sessions, before and after a CSF diverting shunt procedure. The selected cases were retrieved from the Children Hospital Los Angeles (CHLA) repository and presented different flipping angles, resolutions, and averaging schemes. Among the five randomly selected subjects, there is one with normal ventricular characteristics, and the other four presented with at least one abnormality that either deformed the ventricles or created anomalies in intensity and shape that mislead the automatic algorithms while doing the ventricular segmentation. The Table 1 registers acquisition details of each studied subject together with the resulting volumes of manual and automatic segmentations.

**Table 1:** Comparison between manually and automatic extracted volumes. AVVE stands for Automatic Ventricular Volume Estimator.

Item	Resolution	Voxel size (*mm*^3^)	Volume (*mm*^3^)		Difference (*mm*^3^)	Jaccard Index
			Manual	AVVE		
Subject a	(0.41, 0.41, 1.00)	0.17	5065.09	4664.51	400.58	0.87
Subject b	(0.43, 0.43, 1.00)	0.18	5387.72	5036.74	350.98	0.90
Subject c	(0.44, 0.44, 1.00)	0.19	4435.07	3909.47	525.60	0.89
Subject d	(0.45, 0.45, 1.00)	0.20	3001.53	5805.10	-2803.57	0.66
Subject e	(0.58, 0.58, 1.00)	0.37	11383.99	10900.66	483.32	0.89
Mild	(0.62, 0.62, 4.99)	1.95	28238.27	27666.00	572.26	0.92
Moderate	(0.59, 0.59, 4.00)	1.41	271184.35	262207.29	8977.05	0.92
Severe	(0.39, 0.39, 4.99)	0.76	587793.99	581557.78	6236.21	0.94
S1 Before	(0.46, 0.46, 4.00)	0.86	171016.79	154477.01	16539.78	0.91
S1 After	(0.47, 0.47, 5.00)	1.01	129592.70	119572.13	10020.56	0.94

### 2.3 Driving concept

It is often difficult for a programmatic tool to deal with the high degree of variability present in the clinical brain images [17, 18]. This variability includes but is not limited to different image acquisition parameters, the existence of non-natural objects like CSF diverting shunt hardware, general deformation, localized deformation, and positioning constraints. Nevertheless, a minimally trained human can localize and delineate the boundaries of the ventricles even in the worse case scenario where all the hardening factors converge. This complex task is possible because the human can develop identification skills that are not available in any computer system. We artificially reproduce in the computer, the human ability to learn; thus, our methods can deal with the uncertainty in the same manner humans do.

The biggest barrier to the automation is the human visual capability [19] as it is almost impossible to reproduce. We observe objects in space, and with the *a-priori* knowledge of their form; therefore, we can later localize them regardless of the scale, position or surrounding noise [20]. The computer can be fed a structure; however, the computer will have trouble defining if the given structure has been randomly deformed, linearly modified from a given model or is a noisy version of what is being searched. We propose a voxel by voxel analysis that the computer can classify as to whether a given voxel belongs to the searched structure. The composition of the included voxels should yield the form that we wish to segment. For this purpose, we extract features from the images selecting those that create statistical differences between the voxels conforming to our volume of interest versus other voxels in the image.

Naturally, one particular feature is not enough. For instance, the levels of intensity in the ventricular region may also appear in regions of gray matter affected by noise or in empty spaces close to limits of the FOV. But, if a positioning constraint is also given to the programmatic estimator, the power of discrimination is expected to increase. However, some features if wrongly configured, can also negatively affect the estimation.

Among the initially explored features we included: the raw intensity, that is a direct candidate; the Laplacian, that is a boundary detector; filtered versions of the Laplacian using Gaussians with *σ* = [1, 2, 3] as suggested in [17]; and the four features that finally throw the higher score when using the strong-force algorithm presented in Listing 1. Detailed explanation of the four selected features is provided in sections 2.3.1-2.3.4.

In the code provided in Listing 1, the two FOR sentences (lines 5 and 6) run over all permutations among the group of features (feature_ls), that are iteratively selected as columns in the pandas structure (pd_data). The function *selectPermutation* (line 7), returns the needed data to perform the analysis in each grouping and the names of the used features. Note how an SVM is performed in each iteration (line 13). In this feature-selection exercise, we let the *cross_validation* (line 15) function to deal with over-fitting. The index of the highest value in the score array (maxS) returns the best features when used in the participants array (participants[maxS]). This last operation is not listed.

1 def testKbestFeatures(feature ls, pd data):

2   scores = []

3   participants = []

4   regsels = []

5   for n in range (len (feature_ls ) + 1):

6    for r in range (len (feature_ls + 1))

7     ar, lbls, sfeats = selectPermutation (pd_data, n, r)

8     est = feature_selection . SelectKBest (k = r)

9     est. fit(ar, lbls)

10     tregs = est . get_support(indices = True)

11     regsels . append(tregs)

12     ndata = est . transform(ar)

13     estsvm = svm . LinearSVC()

14     gs = grid_search . GridSearchCV(estsvm,{ ‘C’:np . logspace(-4,3)})

15     tscore = np . mean(cross_validation . cross_val_score (gs, ndata,

16          lbls, n_jobs = 6))

17     cores . append(tscore)

18     participants . append(sfeats)

19   return scores, participants, regsels

**Listing 1**. Core code of the strong-force algorithm to select the best features.

The feature-selection exercise detailed above suggested the following features to be the most robust set for automatically segmenting the brain ventricles.

#### 2.3.1 Histogram-based parcellation

A strong characteristic that a human operator uses when attempting to separate structures in a medical image is the contrast. In computer tomography (CT) the contrast is a global characteristic that is derived from the differences in radiation’s absorption of the structures in the FOV [21]; therefore, consecutive imaging intakes lead to similar results [22]. In MRI however, the contrast is generated by differences in spin recruitment that may vary between intakes [23], meaning that each image is made with a different contrast pattern and for that reason, a deterministic threshold would not work in all cases.

**Fig 1:**
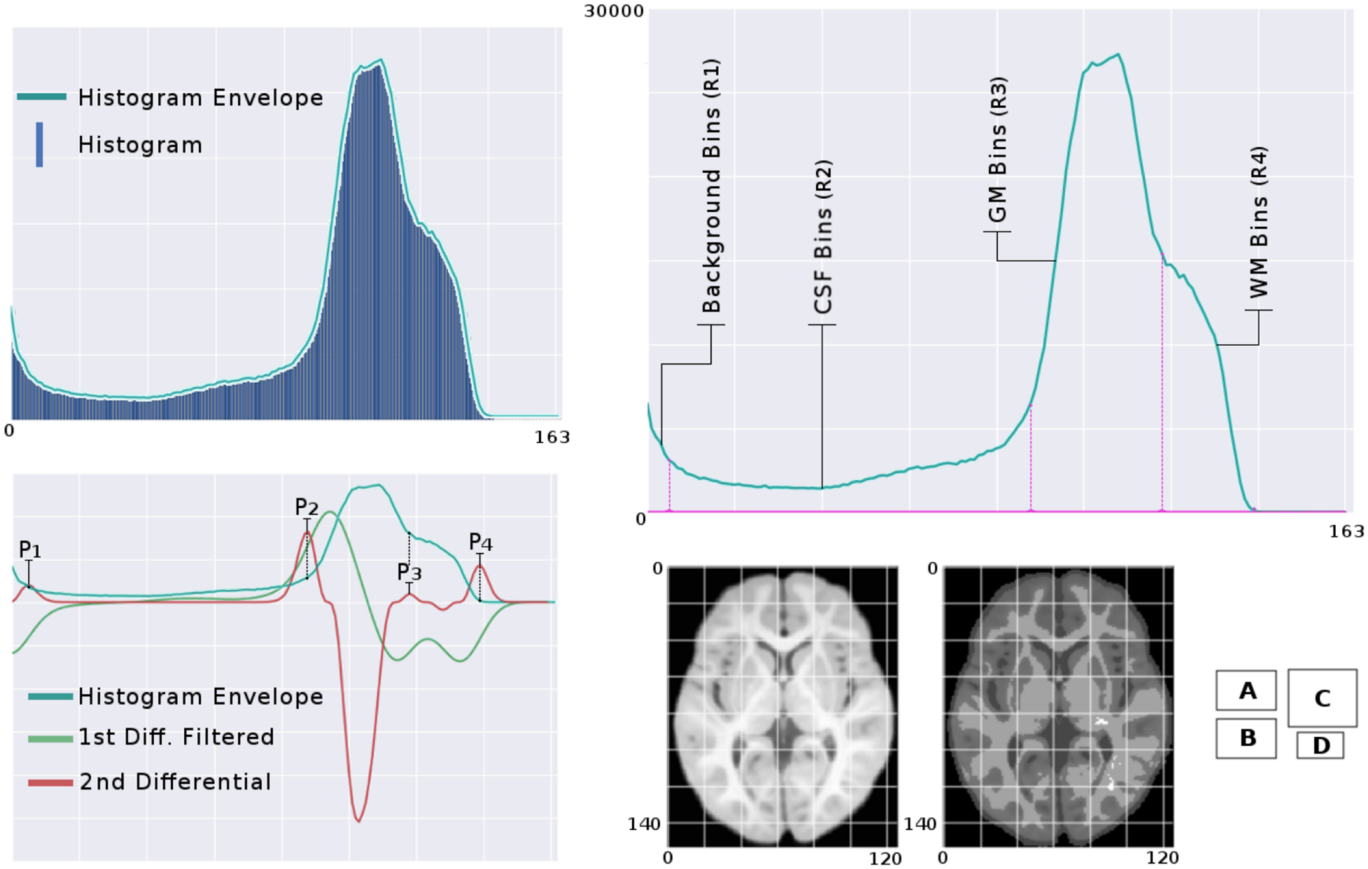
Histogram classified intensity feature. In panel A, the enveloping signal (EnvS) of the histogram is extracted. In panel B. The inflection points of the EnvS are detected using the positive peaks of the power of five of the second derivate. Panel C shows the estimated parcellation in the original EnvS. Panel D shows an axial slice of healthy neonate that presents low contrast (left) and its parcellation (right).

The histogram, Fig 1, captures the intensity distribution of the images and the inflection zones in its envelope estimate the classes present in the image [24]. A second derivate operator detects those inflection zones of the histogram. Due to the different protocols existing in our dataset, some images display a low contrast. Therefore all the derived histograms underwent a signal stretching procedure that not only provides standardization to the process but enhances the detection of slope changes in the signal. Once the inflection points are found, a signal compression is run to return the inflection points to their original position and therefore effect region separation. The outcome of this sub-processing are the labels R1 to R4 that define the boundaries of background (BG), CSF, gray matter (GM), and white matter (WM). The created algorithm uses recursion to identify the four most prominent peaks in a second order derivate signal, that is in turn obtained from the envelope of the histogram. The recursion breaks when four peaks are found. To this end, each iteration adapts the thresholding step of a function applied to the second differentiation array affected by a power-of-five function. This last operation amplifies the significant changes while diminishes the small ones. Also, the even nature of the power-of-five function keeps the polarity of the detected peaks. The positive peaks depict the desired class boundaries.

The extraction of this feature is generalized as follows:

Let *f*(*x*) be the function representing the envelope of the histogram of image *Im*. Then, the positive values of *x* where $\frac{d^{2}f}{dx^{2}}=0$, will pick the abrupt changes in the envelop. The four most prominent detected peaks (*p*_1_, *p*_2_, *p*_3_, *p*_4_) are assigned as the intensity boundaries of each structure in the brain and then, back in the image domain, the voxels intensities *I*(*V*_*i*_) serve to label the each *V*_*i*_ as follows:

$$HCI_{i}=\{\begin{array}{ll} \text{R}1, & \text{if}\;I(V_{i})<p_{1}\\ \text{R}2, & \text{if}\;p_{1}<I(V_{i})<p_{2}\\ \text{R}3, & \text{if}\;p_{2}<I(V_{i})<p_{3}\\ \text{R}4, & \text{if}\;p_{3}<I(V_{i})<p_{4} \end{array}\;\forall \;V_{i}\in Im$$

Thus, every voxel in the image will have a parcellation factor associated due to the position of its intensity in the histogram.

#### 2.3.2 Distances to the center of the magnet

**Fig 2:**
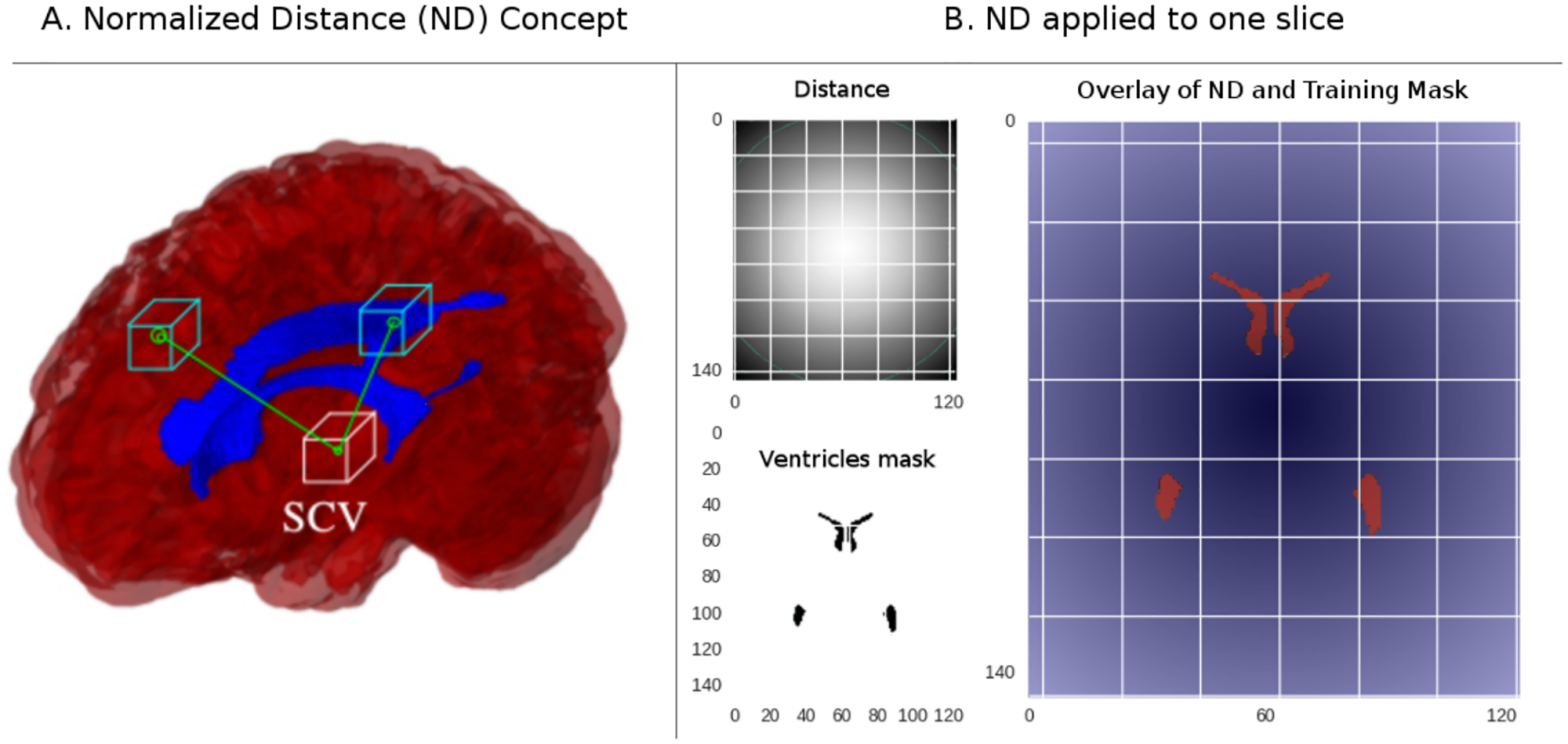
Normalized distances feature. On the left, a figure depicting the ND concept. Every voxel is assigned its distance to the scanner central voxel (SCV). In the figure at the right, we have colored the ND feature creating a fading fashioned effect. Here it is evident how every voxel in and out of the learning masks is overlayed with a different number. Although this feature creates separation, voxels radially equidistant with different intensity value would be difficult to assess for any statistical learning algorithm. Therefore, another feature to eliminate this possible ambiguity is needed.

The distances need to be normalized. In several applications, distances, and other features are normalized by referencing them to a template [25, 26]. When registration takes place, the resulting transformation matrices are used to calculate the real distances in the studied subjects. Here instead, we use the distance normalization strategy proposed in nibabel [27], where the images are referred to the isocenter of the scanner. Therefore, the distance to every voxel to the center of the scanner can be obtained by applying the transformation included in the affine field provided by the nibabel object after an image has been successfully loaded. This procedure does not involve any interpolation; thus, the information used is the one initially obtained in the scanner. See Fig 2 for reference.

Next, each voxel is accompanied by a normalized distance entry which is obtained as follows.

$$\begin{array}{c} ND_{i}=\sqrt{{\left(x_{i}-SCV_{x}\right)}^{2}+{\left(y_{i}-SCV_{y}\right)}^{2}+{\left(z_{i}-SCV_{z}\right)}^{2}}*V_{size}\;\;\forall \;V_{i}\in Im \end{array}$$ (1)

Where *SCV* stands for scanner central voxel, *x*_*i*_, *y*_*i*_, *z*_*i*_ are the coordinates of voxel *V*_*i*_ and *V*_*size*_, stands for voxel size which is obtained as the product of the in-plane resolution factors and the slice thickness.

#### 2.3.3 Cardinality

**Fig 3:**
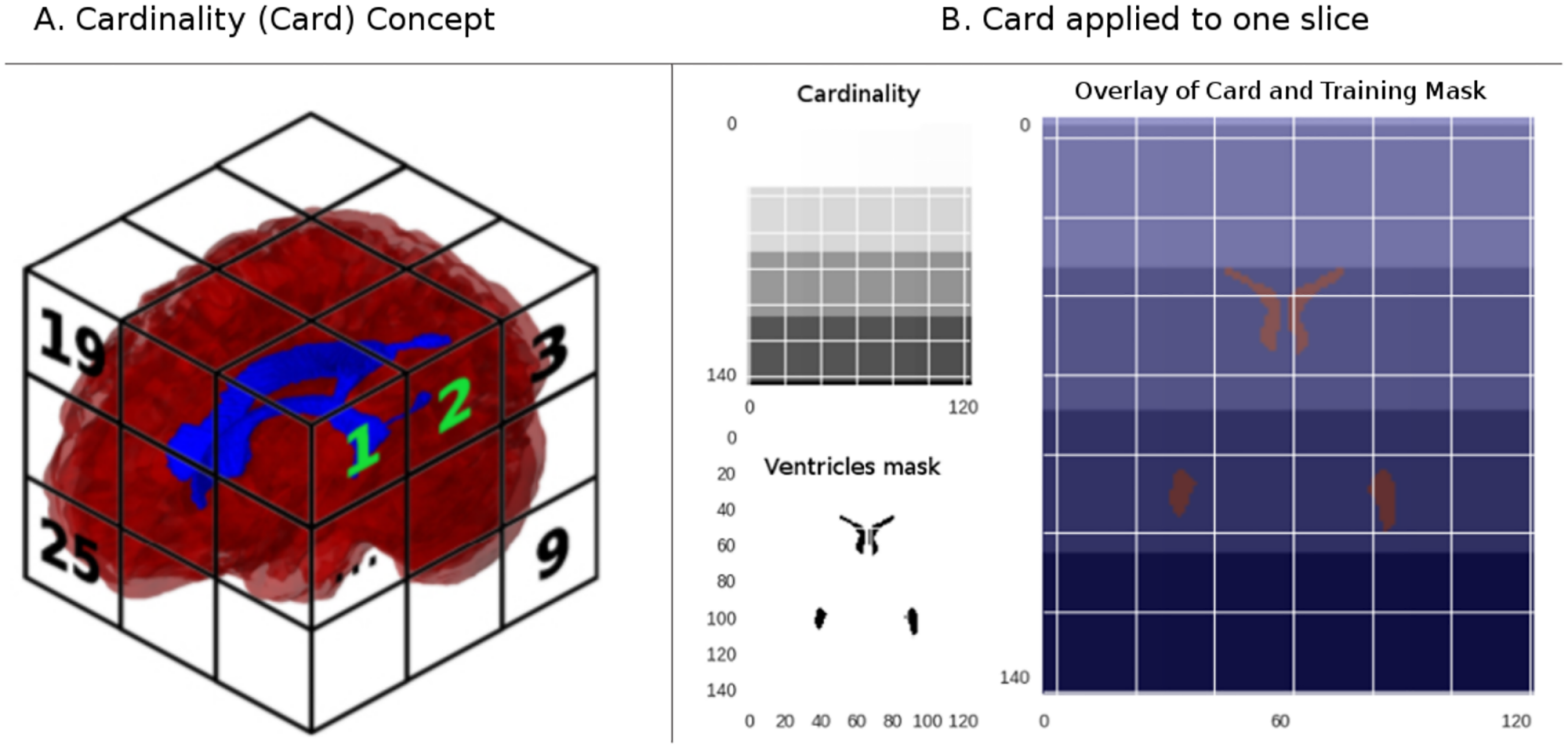
The cardinality feature. In the card feature concept (left), the whole volume is divided into units bigger than a voxel, and each division is assigned a consecutive number. In the card example shown in the right, the grid size has been exaggerated for visualization purposes. Note how there is not only differentiation in the row, but also fading colors at a column level. This FOV demarcation voids the learning discrepancy presented in the ND feature regarding its potential radial ambiguity.

The distances as presented in **Subsection 2.3.2** have a major drawback. They are radially repeated and, if working together with the intensity, the computer can find it difficult to learn that two voxels located at the same distance have different values. This lack of consistency is highly probable due to the asymmetry of the structures inside the brain, especially in the presence of abnormalities. To overcome this issue, we added a third feature to the analysis; the concept of cardinality. In this feature, we divide the FOV in *m* isometric cubes with length size *n*. Later, each sub-cube is assigned a number consecutively incremented in x, then y and finally the z-axes. See Fig 3 for reference.

For the cardinality feature, let *D*_*x*_, *D*_*y*_ and *D*_*z*_ be arbitrary dividers of the image axis. Then, using the length of the axes *R*, let $Div_{x}=\frac{R_{x}}{D_{x}},Div_{y}=\frac{R_{y}}{D_{y}}$ and $Div_{z}=\frac{R_{z}}{D_{z}}$ be the size of segments created with the *D* dividers. With this information in place, the voxels will hold a integer factor given by:

$$\begin{array}{c} card_{i}=\frac{x_{i}}{Div_{x}}+Div_{y}*\left(\frac{y_{i}}{Div_{y}}\right)+{\left(Div_{z}\right)}^{2}*\left(\frac{z_{i}}{Div_{z}}\right)+1\;\forall \;V_{i}\in Im \end{array}$$ (2)

#### 2.3.4 Neighboring

This neighboring feature works like a boundary detector if translated to the image domain. But, computationally speaking, provides every voxel with a knowledge of its 26 neighbors—or less if the voxel is at one limit of the FOV—in its three-dimensional neighborhood. The mechanism defines a direction starting from the voxel under study that points to one of its intimate neighbors (*V*_*n*_), the intensity of this pixel is saved. Then, the analysis is propagated to one more voxel in the selected direction to a second voxel which is the neighbor (*V*_*nn*_) of the initially selected voxel (*V*_*n*_). Then, if the differential of intensity between *V*_*n*_ and *V*_*nn*_ is 20% greater from the one between the current voxel and (*V*_*n*_), the (*V*_*n*_) won’t be considered a neighbor. In case *V*_*n*_ must be labeled as a neighbor, a counter is incremented; therefore, this index is framed between 0 and 26 for any voxel in the image, excepting the ones on the border of the FOV.

**Fig 4:**
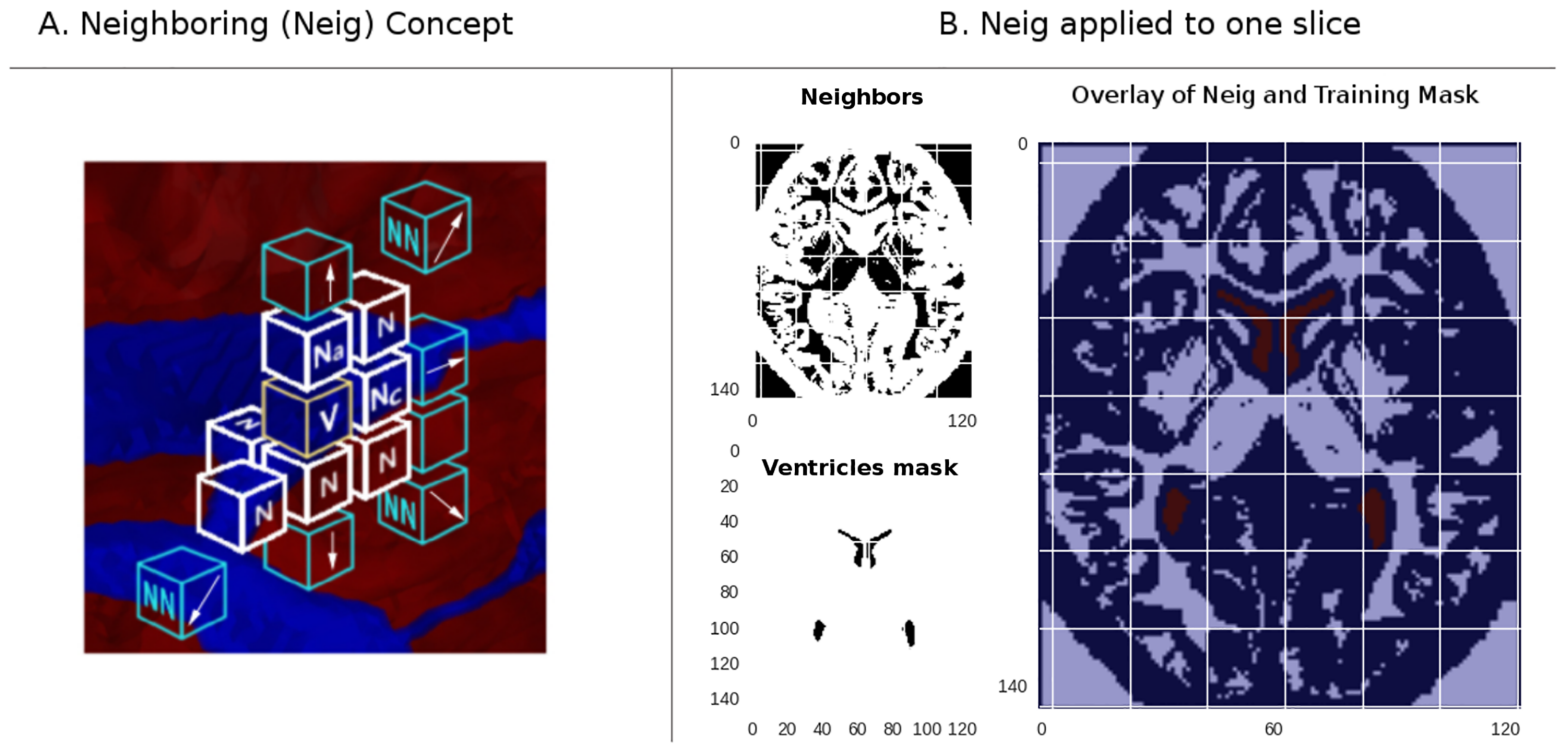
The neighboring feature. The Neig concept (left) creates boundaries where the voxels are too different in intensity. In the normal brain (right), its contribution is nil. However, in case of abnormalities or the presence of other elements inside the ventricles such a CSF diverting shunt, this feature provides meaningful information.

Refer to Fig 4 for the neighboring feature generalization explained below. Assume that voxel *V* is in a position given by coordinates (*x*, *y*, *z*). Assume also that each coordinate can be independently varied with the values in array *step* = [−1, 0, 1]. This allows the system to query the intensity of all voxels *V*_*N*_. Also, when querying the intensity of a *V*_*N*_ reached with step = [a,b,c], the *V*_*NN*_ is located at (*V*_*Nx*+*a*_, *V*_*Ny*+*b*_, *V*_*Nz*+*c*_).

The outputs in the following expression are saved in the array *c*.

$$c_{k}=\{\begin{array}{ll} 1, & \text{if}\;abs(I(V)-I(V_{N}))>0.2*(abs(I(V_{N}-I(V_{NN})))\\ 0, & \text{otherwise} \end{array}\;\forall step!$$

And then,

$$\begin{array}{c} Neig_{i}=\sum^{k}c_{k}\;\forall \;V_{i}\in Im \end{array}$$ (3)

Recall that *step* = [0, 0, 0] is an exception in the formulation above because it points to the voxel under test.

#### 2.3.5 The statistical estimator

**Fig 5:**
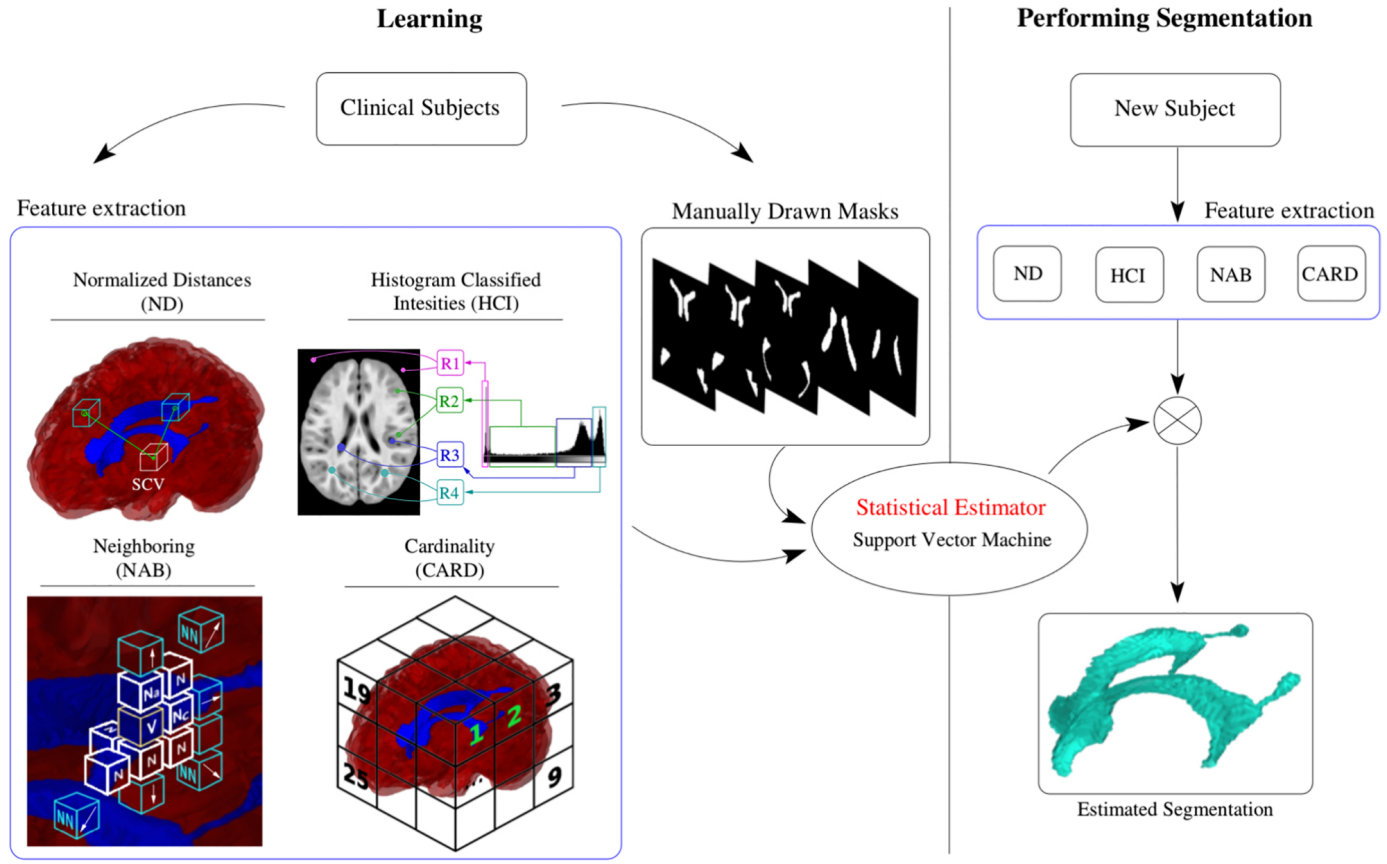
Learning and performing the segmentation processes. The features are extracted for each voxel in the training images, and a support vector machine creates the separating hyperplane. Once the separating hyperplane is created, the automatic segmentation in any new subject is performed by extracting the same features used in the training process and the statistical estimator.

The four features explained in sections 2.3.1 through 2.3.4, produce a number with their span. This information is scaled using the method *StandardScaler* of the class *preprocessing* available in scikit-learn. In all stages where needed, the data is fed by its reference to the subject e.g. in the estimator creation, data is separated 0.75:0.25 (training: testing). The information is separated by subject. Therefore in each iteration of the 6-folding experiment, the information of 33 subjects is used as the training dataset, and 11 subjects are used as testing data. With this particular manner of gathering the information we avoid the problem of feeding the algorithm with data that do not represent the whole segmenting structure, which is possible when working in a voxel-based fashion. Once the estimator is created, we abandon the training stage and proceed to the classification that yields the automatic ventricular separation. The manually segmented masks of the 10 tested cases are also provided to support the comparison scheme presented in the Table 1. Fig 5 conceptualizes the creation and use of the statistical estimator.

The CHLA IRB approved the use of this retrospective data, and all the health insurance portability accountability act (HIPPA) directives were carefully followed during the development of the hypothesis.

## 3 Results

**Fig 6:**
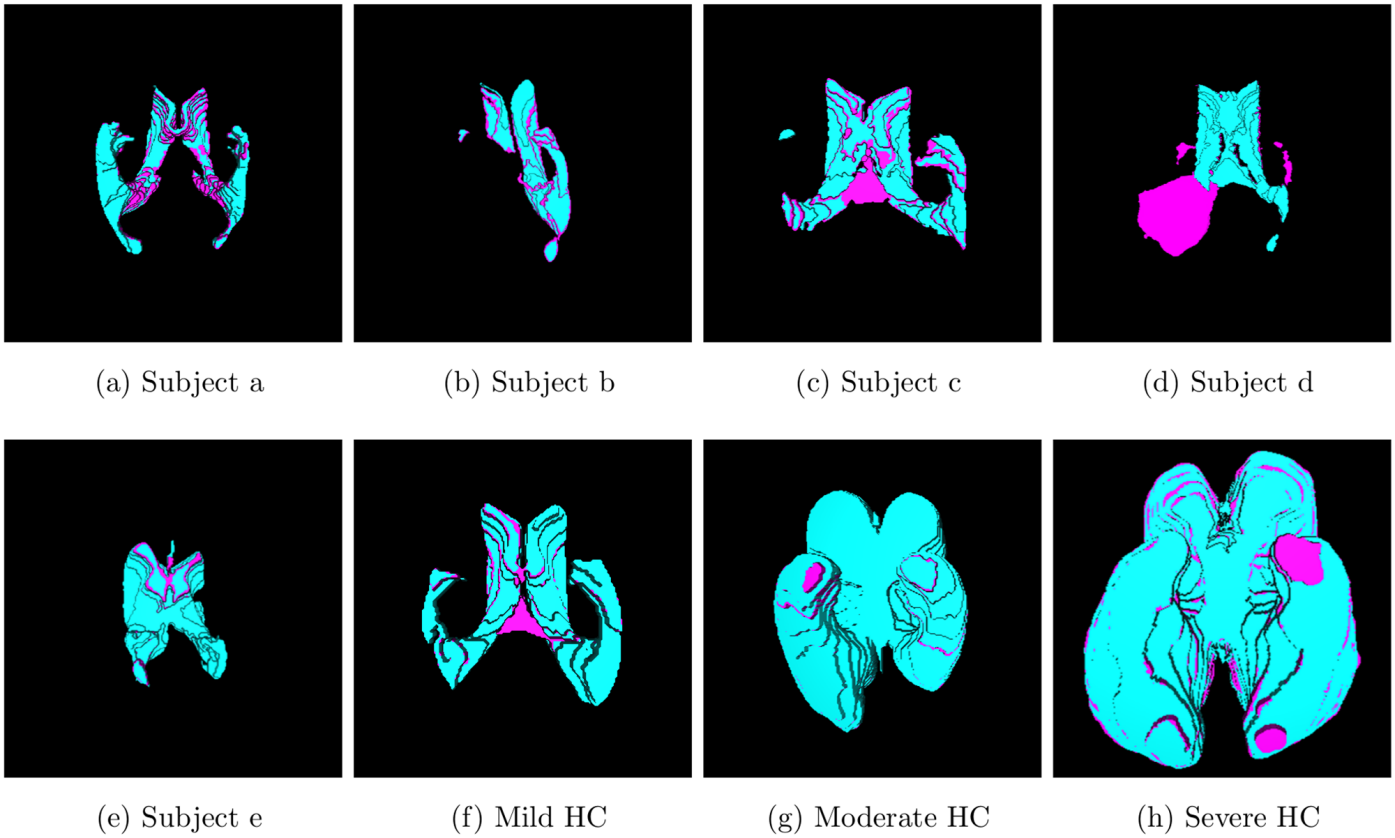
Overlapping between manual (cyan) and AVVE (magenta) segmentations. These axial views obviate big portions of the surface. For a complete reference, see the companion video. The large discrepancy in (d) is secondary to an arachnoid cyst containing CSF adjacent to the occipital horn of the right lateral ventricle.

Manual segmentations of the ten studied images selected were performed for comparison with the automatic ventricular volume estimator (AVVE). In this comparison, the volume differences and the Jaccard index [28] are used. The volume differences define the overall discrepancies between manual and automatic assessments. The Jaccard index says how well the two compared structures overlap. When compared with its manual counterpart, the AVVE underestimated the volumes in all cases, except in the one where an arachnoid cyst adjacent to the ventricles misled the algorithm, see panel d in Fig 6.

**Fig 7:**
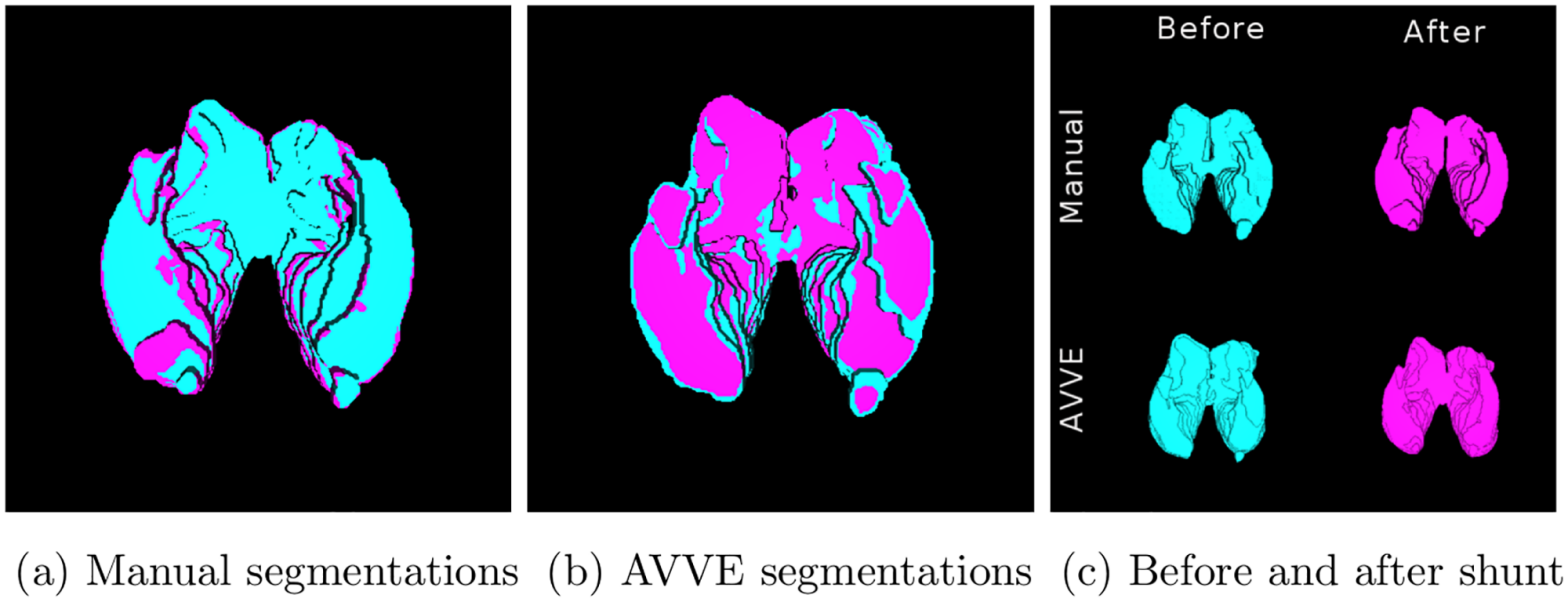
Before and after shunt procedure masks. Manual (cyan) and AVVE (magenta) delineations are overlapped. See companion video for a 360° visualization.

See on Fig 7, the results of the manual and automatic segmentations for subject *S*1 in Table 1, that was scanned before and after a shunting procedure.

## 4 Discussion

The machine learning implementation proposed here reached a 94% of accuracy and it holds promises to improve further in the light of the unlimited amount of available data in the PACS with which to learn. Also, other meaningful features can be considered in the future, including those associated with age factors that are available in the DICOM headers within the PACS [29].

An arachnoid cyst (AC) adjacent to the segmenting structure results in subject d having a low Jaccard index. The AC appears in the boundary of the segmenting structure thus some of the voxels in the AC are read as part of the lateral ventricles. The CSF within the AC appears the same as CSF in the ventricles and subarachnoid space. The reason why the algorithm failed is due to the absence of AC samples in the training data. In other words, we have not designed the instrument to deal with this rare occurrence.

The incidence of an AC within the central nervous system is approximately 2-3% but only those AC within or adjacent to the ventricles would mislead the segmentation process and constitute a fraction of 1% of all AC [30]

Current solutions that can be classified as registration-based [5, 6] cannot be clinically implemented since they require a minimum-of-structuralism present in the treated images to extract a transformation matrix that links the analytics done in the template with the subject under study. This minimum-of-structuralism requirement may not be fulfilled in clinical studies, where very often the FOV is chosen to select only a particular region of the whole organ.

Regarding thresholding methods such as [31], they have not proved to be fully adaptable to the point of being launched alone as an absolute solution. They are usually presented together with other strategies like in [10, 32]. Moreover, these algorithms have not be reported to be useful clinically or in the presence of abnormalities. Other authors have also proposed voxel-based approaches similar to the one we detail in this manuscript. In [11] segmentation is asserted by a k-nearest neighbor (kNN) classifier that uses spatial information and voxel intensities from different image modalities the availability of which is limited. In [13], it uses a probabilistic atlas to create initial labels in the voxels. The labeling process requires a registration step which is unattainable in the PACS. In [15], an atlas-based kNN classifier is used to perform segmentation using multi-modal MRI data. Here both, the of atlases and multi-modality schemes preclude clinical applicability.

The authors in [12] use a three-layered segmentation procedure where each layer has its classifier. The data consists of images acquired with sedation and reportedly uses the same acquisition protocol regarding not only as to modality but also to spatial resolution. In [12] two structural images are required (T1 and T2), but may not always be available together.

Finally [16] presents a work that uses a one-pass SVM classifier similar to the one we implemented in our solution. However, the solution requires registration and is multi-modality.

One of the most useful aspects of our design is the capacity to extract the necessary features from a single modality image and proceed without the need of any external information, avoiding the need for registration.

Recent sophisticated methods have been presented where the preliminary evidence suggests sufficiency for solving the problem such as [33, 34]; however, are unlikely to be implemented clinically due to their lack of being automatic and need for human interaction.

The presented solution is seen as a plug-in for the PACS platform previously developed [4]. As stated in the abstract, our PACS implementation gives access to images at a bit level so that quantification and analysis can be accomplished. But this opens a significant confidentiality concern. HIPPA laws will be broken if humans interact with the images at bit-level through a graphical user interface mostly because in the network, each unit of information has headers that allow easy identification and association to the individual being imaged. With the automatic nature of the AVVE algorithm, we avoid all these issues. From a final user perspective, the analytics is done by the same storing platform, and no human interaction is needed, except to link the studies and the analytical procedures to be run.

The capability to learn makes the presented strategy available for a more comprehensive purpose, hypothetically accomplishing segmentations in any part of the human body and in the presence of any abnormality.

## 5 Conclusion

A common clinical neurological problem is optimally managing hydrocephalus for which many MR/CT studies are done to determine if the ventricular volume has changed. It is anticipated that soon, the neuroradiology report will routinely include a number indicating ventricular volume. This technique can be applied to any cerebral structure that can be segmented such as GM,WM, various nuclei, tumors, aneurysms, etc. Treatment effect can be better monitored and characterized if one is able to measure any volume change accurately.

## Supporting information

S1 Video A set of 360° visualizations with overlapped manual and AVVE ventricular segmentations. The video complements the images presented in Figs 6 and 7.
(MP4)
